# Plant cell culture technology in the cosmetics and food industries: current state and future trends

**DOI:** 10.1007/s00253-018-9279-8

**Published:** 2018-08-11

**Authors:** Regine Eibl, Philipp Meier, Irène Stutz, David Schildberger, Tilo Hühn, Dieter Eibl

**Affiliations:** 1 Institute of Chemistry and Biotechnology, Zurich University of Applied Sciences (ZHAW), 8820 Wädenswil, Switzerland; 2 Institute for Food and Beverage Innovation, ZHAW, 8820 Wädenswil, Switzerland

**Keywords:** Bioreactor, Cellular agriculture, Cosmetic supplement ingredients, Foodstuff and food ingredients, Plant cell culture extracts

## Abstract

The production of drugs, cosmetics, and food which are derived from plant cell and tissue cultures has a long tradition. The emerging trend of manufacturing cosmetics and food products in a natural and sustainable manner has brought a new wave in plant cell culture technology over the past 10 years. More than 50 products based on extracts from plant cell cultures have made their way into the cosmetics industry during this time, whereby the majority is produced with plant cell suspension cultures. In addition, the first plant cell culture-based food supplement ingredients, such as Echigena Plus and Teoside 10, are now produced at production scale. In this mini review, we discuss the reasons for and the characteristics as well as the challenges of plant cell culture-based productions for the cosmetics and food industries. It focuses on the current state of the art in this field. In addition, two examples of the latest developments in plant cell culture-based food production are presented, that is, superfood which boosts health and food that can be produced in the lab or at home.

## Introduction

In 1902, the Australian botanist Gottlieb Haberlandt provided the basis for the usage of plant cell and tissue cultures (Haberlandt 1902). He described the formation of callus (unorganized cell mass in response to wounding) from adult plant cells and its regeneration into a complete plant for the first time. This phenomenon, also known as cellular totipotency of plant cells, was experimentally demonstrated by growing carrot cells in vitro *by* Haberlandt in 1958 (Fehér 2015). Between the 1960s and the 1980s, many studies were executed in order to mass propagate plant cell cultures and to develop bioprocesses delivering secondary metabolites for the pharmaceutical, food, and cosmetics industries. Different commercial secondary metabolites (e.g., shikonin, scopolamine, protoberines, rosmarinic acid, ginseng saponins, and immunostimulating polysaccharides), which are based on plant cell cultures, entered the market between the early 1980s and late 1990s (Sato and Yamada 1984; Deno et al. 1987; Ritterhaus et al. 1990; Hess 1992; Hibino and Ushiyama 1999). A further milestone in plant cell culture technology is represented by the FDA (Food and Drug Administration) approval of the anticancer compound paclitaxel in 2000. Cells from the Pacific yew grown in 75 m^3^ stirred bioreactors deliver up to 500 kg of this medicinally important secondary metabolite per year (Imseng et al. 2014; Steingroewer 2016). The advantages of the production of secondary metabolites with plant cell cultures over conventional agricultural production with whole plants are indisputable (Hussain et al. 2012). There is no seasonal dependence on in vitro production of secondary metabolites, and a controlled manufacture via standardized batches is possible. Furthermore, the impact on the ecosystem is low, the water needed and carbon footprint are reduced, and pesticides as well as herbicides are not required. Nevertheless, the number of commercial production processes of secondary metabolites involving plant cell cultures is low. This particularly concerns pharmaceutical applications and is ascribed to somaclonal variations of the production clones as well as too low secondary metabolite titers (Sharma et al. 2014).

Product approval in the pharmaceutical industry differs from that in the cosmetics industry, where no official approval is required and where the manufacturing company is responsible for product safety (Zappelli et al. 2016). Moreover, innovations and developments in the cosmetics industry, which introduces hundreds of new cosmetics products every year, are strongly driven by the consumer. The consumer wants to have not only effective, safe, and natural but also sustainable, cosmetics products, whose manufacture does not negatively affect the environment (Schmidt 2012; Fonseca-Santos et al. 2015). In respect of the cosmetics industry, there is high interest in plant cell culture extracts with multiple specific activities for skin care, make-up, and hair care as supplement ingredients. Plant cell culture extracts containing a mixture of bioactive ingredients (and not only secondary metabolites) can already be produced under controlled conditions. Moreover, even extracts from rare or endangered plant species can be made available by applying plant cell culture technology. It is also worth mentioning that plant cell culture extracts can be used in minimal concentrations in the final cosmetics formulations (Barbulova et al. 2014). In other words, a low product titer is less critical than in pharmaceutical applications, especially since the plant cell culture extract may act in a synergistic manner as described by Carola et al. (Carola et al. 2012). Consequently, the large number of cosmetics products which have been manufactured with plant cell culture technology over the past 10 years is hardly a surprise. Indeed, it explains the renaissance in plant cell culture technology that has taken place.

The developments in the cosmetics industry have influenced the food industry, where new manufacturing methods for food and food ingredients are also in demand. Various studies have reported that supplying the world population with both animal and plant-based food in sufficient quantity and quality will become increasingly difficult. For example, according to the estimates of Alexandratos and Bruinsma, 60% more food will be required by 2050 than is manufactured today (Alexandratos and Bruinsma 2012), and traditional farming will not be able to meet these requirements. Cellular agriculture is assumed to be one solution here (Foussat and Canteneur 2016; Mattick 2018; Nordlund et al. 2018). Plant cell-based cellular agriculture uses plant cell cultures to manufacture high-value food ingredients (Stafford 1991; Fu et al. 1999; Ravishankar et al. 2007; Nosov 2012; Davies and Deroles 2014). Ginseng triterpene saponins manufactured with plant cell cultures in bioreactors have been used as food supplement ingredients for a considerable time (Wu and Zhong 1999; Sivakumar et al. 2005; Paek et al. 2009), but many plant cell culture lines producing food supplement ingredients have not reached commercial production. Due to the latest approaches to engineer homogeneous and high-productivity cell lines without genetic engineering (Yun et al. 2012; Sood 2017), plant cell culture technology for food products is regaining interest. Climate change and plant diseases reducing the production of plant-based food are driving this trend, and first scientific studies have suggested that plant cell cultures or their extracts may themselves be used as foodstuffs (Räty 2017; Nordlund et al. 2018).

This mini review describes the current state of plant cell culture technology aimed at products for the cosmetics and food industries. Based on an overview of plant cell culture extracts which have been launched by European and US companies over the past 10 years, we present the main plant cell culture types of interest, their establishment, the mass propagation in bioreactors, and the related challenges. In addition, the production of plant cell culture extracts following the bioreactor cultivation step is briefly discussed. Finally, two examples of the latest developments in plant cell culture-based food production are given. However, plant cell culture-based manufacture of recombinant proteins (Tschofen et al. 2016) is not covered, as the vast majority of cosmetics and food companies, particularly those located in Europe, do not use genetically modified plant cell and tissue cultures, which are the precondition for the production of recombinant proteins with plant cell culture technology.

## Product overview of plant cell culture extracts for applications in the cosmetics and food industries

In 2008, Mibelle Biochemistry laid the cornerstone for the successful course of plant stem cell culture extracts into the cosmetics industry. The company launched PhytoCELLTECH *Malus domestica* (Schmid et al. 2008; Schürch et al. 2008; Imseng et al. 2014), the first commercially available plant cell culture extract whose effect was studied on human skin cells and which claims to be derived from plant stem cells. PhytoCELLTECH *Malus domestica* was established from the core of an endangered Swiss apple variety, the Uttwiler Spätlauber, which can be stored for a long time without becoming shriveled or losing flavor. The company has patented the manufacture and usage of apple cell culture extracts which originate from *Malus domestica* cultivar Uttwiler Spätlauber and which protect skin cells (Blum et al. 2013). The manufacture includes cell culture establishment, their cultivation from shake flasks up to the production bioreactor (50–100 L), and liposomal extract manufacture by applying high pressure homogenization. PhytoCELLTECH *Malus domestica* entails numerous plant cell culture extracts which are used by leading cosmetics brands such as Dior, Lancôme, Guerlain, and La Prairie in their cosmetic formulations. The final products include facial serums, facial creams and facial masks, eye creams, make-up products, hair oils, hair serums, and hair conditioners. Table 1 contains a selection of plant cell culture extracts which are important for the cosmetics industry and which contain, for example, polyphenols, vitamins, fatty acids, peptide mixtures, and saccharides. They have been successfully brought to market by European and US companies over the past 10 years. The prevailing majority of these product candidates have “stem cell” in their product name. It indicates that the plant cell culture extract originates from plant meristems such as a shoot apical system, root apical system, or cambium (Greb and Lohmann 2016). In Table 1, plant cell culture-based food supplement ingredients and their manufacturers are also shown. Teoside 10 was the first plant cell culture extract approved as a food supplement ingredient in Europe (Dal Toso and Melandri 2010; Dal Toso and Melandri 2011a; Dal Toso and Melandri 2011b). Recently, the food supplement ingredients Acetos 10P, Teupol 10P, Teupol 50P, and Echinan 4P have been authorized as Novel Food according to Article 5 of the EU Regulation 258/97 (Fremont 2017).

**Table 1:** Plant cell culture extracts manufactured by European and US companies which have entered the market in the cosmetics and food industries during the past 10 years. Asian manufacturers are not considered. The manufacturer’s names are listed in alphabetic order (no rating). The list of products makes no claim to be complete

Product	Plant species	Application	Manufacturer	Reference
Acetos 10P	*Lippia citriodora*	Food: supplement ingredient	Active Botanicals Research (ABR) www.abres.it	Fremont (2017)
Teupol 10P and Teupol 50P	*Ajuga reptans*			
Echinan 4P	*Echinacea angustifolia*			
Celtosome	*Crithmum maritimum*	Cosmetics: skin rejuvenation and care	BiotechMarine by Seppic www.seppic.com/seppic/biotechmarine	John (2017)
	*Eryngium maritimum*			
Cocovanol^1^	*Theobroma cacao*	Food: supplement ingredient	Diana Plant Sciences^2^ www.diana-food.com; www.symrise.com	Barney (2013), Georgiev (2015)
Plant C-Stem *Vigna radiata*	*Vigna radiata*	Cosmetics: skin rejuvenation and care	innovacos www.innovacos.com	Khan (2017)
Stem cell extracts from: roseroot, greater plantain, milk thistle, *Aloe vera*	*Rhodiola rosea*, *Plantago major*, *Silybum marianum*, *Aloe barbadensis* Mill.		In vitro Plant-tech www.invitroplanttech.se	Bengtson (2018)
Flower Power Innovation extract	*Calendula officinalis* and *Silybum marianum*			
Stems GX products: Buddleja Stems GX, Echinaceae Stems GX, Gardenia Stems GX, Lenontopod Stems GX, Resistem, Marubium Stems GX	*Buddleja davidii*, *Echinacea angustifolia*, *Gardenia jasminoides*, *Leontopodium alpinum*, *Globularia cordifolia*, *Marrubium vulgare*		Institute of Biotechnological research (IRB) by Sederma^3^ www.sederma.fr); (www.irbtech.com; www.croda.com	Dal Toso and Melandri (2010), Schäfer (2012)
Stems GX products: Centella Stems GX	*Centella asiatica*	Cosmetics: treatment of rosacea	Institute of Biotechnological research (IRB) by Sederma^3^ www.sederma.fr; www.irbtech.com; www.croda.com	Dal Toso and Melandri (2011a), Dal Toso and Melandri (2011b), Unknown (2013)
Dermasyr 10	*Syringa vulgaris* (lilac)	Cosmetics: treatment of acne and sebum-related disorders		
Echigena plus	*Echinacea angustifolia*	Food: supplement ingredient		
Teoside 10	*Ajuga reptans*			
ReGeniStem Brightening	*Glycyrrhiza glabra*	Cosmetics: skin brightening	Lonza www.lonza.com	Lonza (2014)
Stem cell culture extract from arctic cloudberries	*Rubus chamaemorus*	Cosmetics: skin rejuvenation and care	Lumene www.lumene.com	Nohynek et al. (2014); Suvanto et al. (2017)
PhytoCELLTECH actives from: argan tree, apple, alpine rose, soapwort, comfrey and grapes	*Argania spinosa*, *Malus domestica* (Uttwiler Spätlauber), *Rhododendron hirsutum*, *Saponaria pumila*, *Symphytum officinale*, *Vitis vinifera* (Gamay Teinturier Fréaux)		Mibelle Biochemistry www.mibellebiochemistry.com	Schmid et al. (2008), Schmid and Zülli (2012), Schmid et al. (2013), Imseng et al. (2014), Morus et al. (2014), Trehan et al. (2017)
RootBioTec HO	*Ocimum basilicum*	Cosmetics: treatment of hair loss		Belser (2015)
Callus stem cell extracts from: orchid, lotus, tomato, rice, grape, carrot, green tea, ginseng	*Neofinetia falcata*, *Nelumbo nucifera*, *Solanum lycopersicum*, *Oryza sativa*, *Vitis vinifera*, *Daucus carota*, *Camellia sinensis*, *Panax ginseng*	Cosmetics: skin rejuvenation and care	Sandream Enterprises www.sandreamimpact.com	www.ultraspector.com. retrieved on May 11, 2018
Stem cell culture extracts: BerryFlux Vita, Cell integrity, Bionymph peptide, Cell Pulse, Daphne VitaSense, FicuCell Vita, Hibiskin Vita, Lykosin defense, Vita Freeze, Mythus Vita, Vita Nova, VitaShape, VitaLight	*Rubus idaeus*, *Nicotiana sylvestris*, *Psilanthus bengalensis*, *Daphne odora*, *Opuntia ficus-indica*, *Hibiscus syriacus*, *Solanum lycopersicum*, *Actinidia deliciosa*, *Lotus japonicus*, *Coleus forskohlii*, *Cirsium eriophorum*	Cosmetics: skin rejuvenation and care, skin brightening and firming	Vitalab www.vitalabactive.com	Apone et al. (2010), Barbulova et al. (2010), Bimonte et al. (2011), Tito et al. (2011), Tito et al. (2015), Di Martino et al. (2017)
Plasma Rich in Cell Factors (PRCF) products: Arabian Cotton, Luminia Granatum, Sensia Carota	*Gossypium herbaceum*, *Punica granatum*, *Daucus carota sativa*	Cosmetics: skin rejuvenation and care, skin whitening	Vytrus Biotech www.vytrus.com	Juanis (2017)
Phyto-Peptidic Fractions (PPF) products: Capilia Longa	*Curcuma longa*	Cosmetics: treatment of hair loss		
PPF products: Centella Reversa	*Centella asiatica*	Cosmetics: skin rejuvenation and care		

^1^No longer available

^2^Now part of Symrise AG

^3^Member of the Croda International Group

## Applied plant cell culture types

### Cell suspension cultures from dedifferentiated callus cells and undifferentiated cambial meristematic cells

**Fig. 1:**
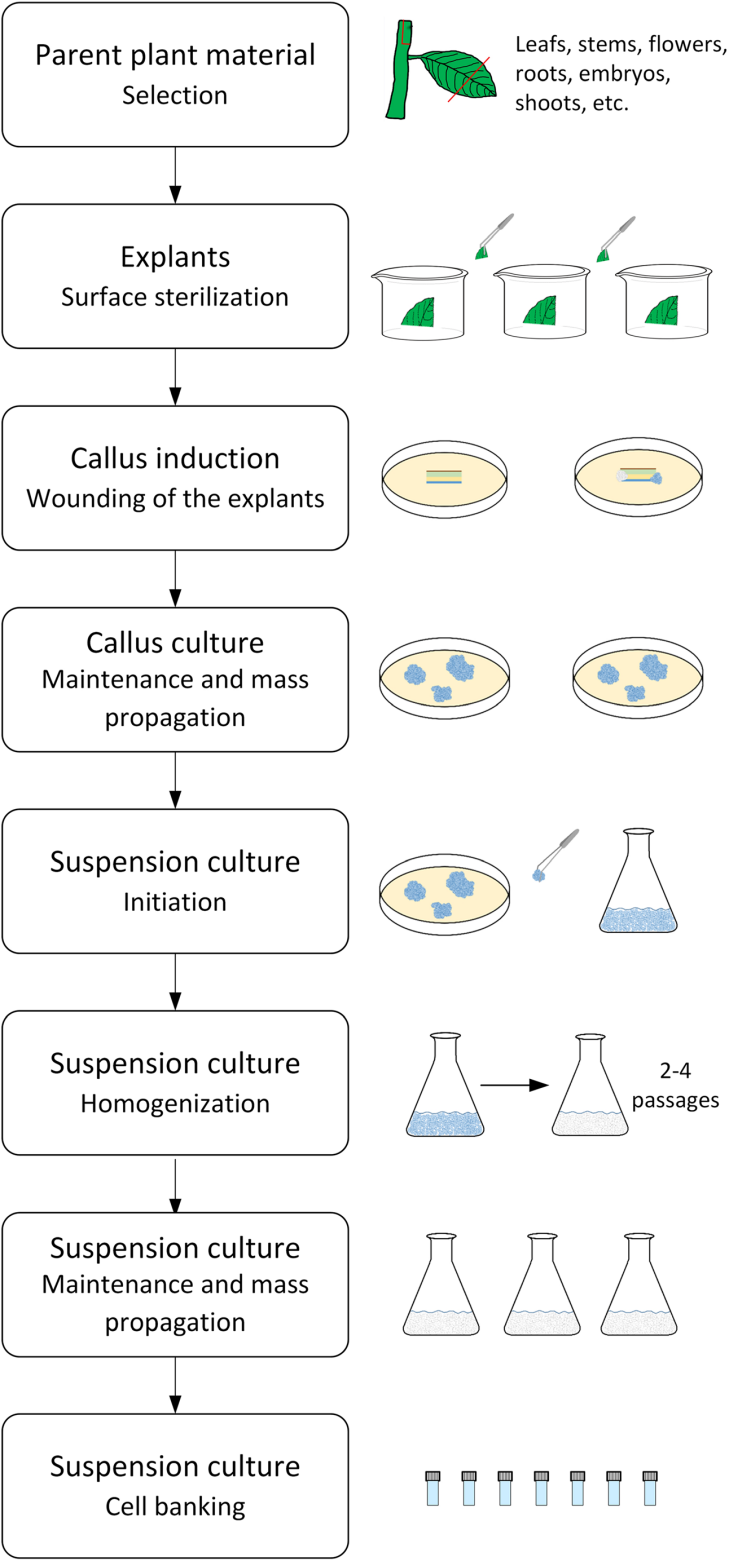
Schematic representation of the procedure for the establishment of a DDC-based plant cell suspension culture

The plant cell culture extracts are most frequently derived from cell suspension cultures that have been created from dedifferentiated plant cells (DDCs). The common modus operandi to establish a DDC-based plant cell suspension culture is illustrated in Fig. 1. The main steps of the procedure include the selection of potent parent material, an optimized surface sterilization procedure, the induction, maintenance and mass propagation of the callus culture in petri dishes, the initiation, homogenization, maintenance and mass propagation of the suspension culture in shake flasks and bioreactors, and the final cell banking of the suspension production cell line. Although all parts of a plant can be used to initiate a callus culture, it is important to select the most suitable parent plant and organ type which contains the bioactive compound(s) of interest in the desired quantity and quality. The quantity and quality of the bioactive compound(s) of interest are greatly affected by the plant species, its development stage and location, and the plant organ (also referred to as explant) type. Growth regulators (phytohormones which are a combination of auxins and cytokinins) that are added to the culture medium also have to be taken into account when establishing a high performing callus culture that is friable, grows and produces well, and is stable. Both type and concentration of the growth regulators have an influence on callus growth and morphology as well as on secondary metabolite synthesis (Evans et al. 2003; George et al. 2008; Gutzeit and Ludwig-Müller 2014). Thus, a high work load is already required to identify the most promising candidates among the different callus cell lines to be initiated, maintained, and mass propagated. After the callus cell line selection, the suspension cell culture is generated, as exemplarily presented in Fig. 1. Calli are transferred from a petri dish into a shake flask containing liquid culture medium. With subsequent cultivation in a shaker, the size of the callus cell aggregates becomes smaller. This improves mass transfer by increasing the specific growth surface and implicates a higher growth velocity of cells in the shake flask than in the petri dish. A successive homogenization procedure as described by Eibl et al. (2009a) reduces the time needed to provide a homogeneously growing and producing plant suspension culture. Furthermore, it is important to mention that the culture medium is often modified for maintenance, growth, and production in terms of phytohormone type and concentration, and levels of nitrogen, phosphate, and sucrose (Bhojwani and Dantu 2013; Murthy et al. 2014). This is done with Murashige and Skoog (Murashige and Skoog 1962), Schenk and Hildebrandt (Schenk and Hildebrandt 1972), or Gamborg B5 medium (Gamborg et al. 1968), for example. DDC-derived plant suspension cells reaching typical doubling times of between 2 and 4 days normally grow in aggregates that consist of up to hundreds of cells. The aggregate formation, mainly ascribed to the formation of extracellular polysaccharides in older cultures, may change the rheology of the culture broth, limit mass transfer, and reduce cell growth and product formation (Eibl et al. 2009a). Moreover, with increasing cultivation time, genetic instabilities of the DDCs may occur, caused by somaclonal variations and evidenced by decreased or complete loss of product formation (Georgiev et al. 2009). The availability of a working cell bank containing the cryopreserved, DDC-derived production suspension cell line reduces the risk of somaclonal variations because of reduced subcultivation intervals. As in the case of mammalian suspension cultures, the controlled rate slow freezing approach is the gold standard for DDC-based plant suspension cells (Lawrence 2015; Schumacher et al. 2015). However, in comparison to mammalian cells, cell regrowth is more difficult for plant cells after thawing.

Due to the advantages of undifferentiated cambial meristematic cells (CMCs) over DDCs, CMC-based plant suspension cultures have gained increasing attention over the past few years (Lee et al. 2010; Lee et al. 2012; Moon et al. 2015; Ochoa-Villarreal et al. 2015; Sood 2017). CMCs, which have small spherical abundant vacuoles, are morphologically and physiologically stable, grow as single cells, and are easy to regrow after cryopreservation. Homogenization procedures are becoming obsolete, and CMC-based suspension cultures have a superior growth and production performance to that of DDC-based ones. The company Unhwa, owner of the world’s first patent for CMC isolation and cultivation, successfully developed CMC-based suspension cultures of *Taxus cuspidata*, *Ginkgo biloba*, and *Solanum lycopersicum* for applications in the cosmetics industry (Loake and Ochoa-Villareal 2017).

### Plant tissue cultures

**Fig. 2:**
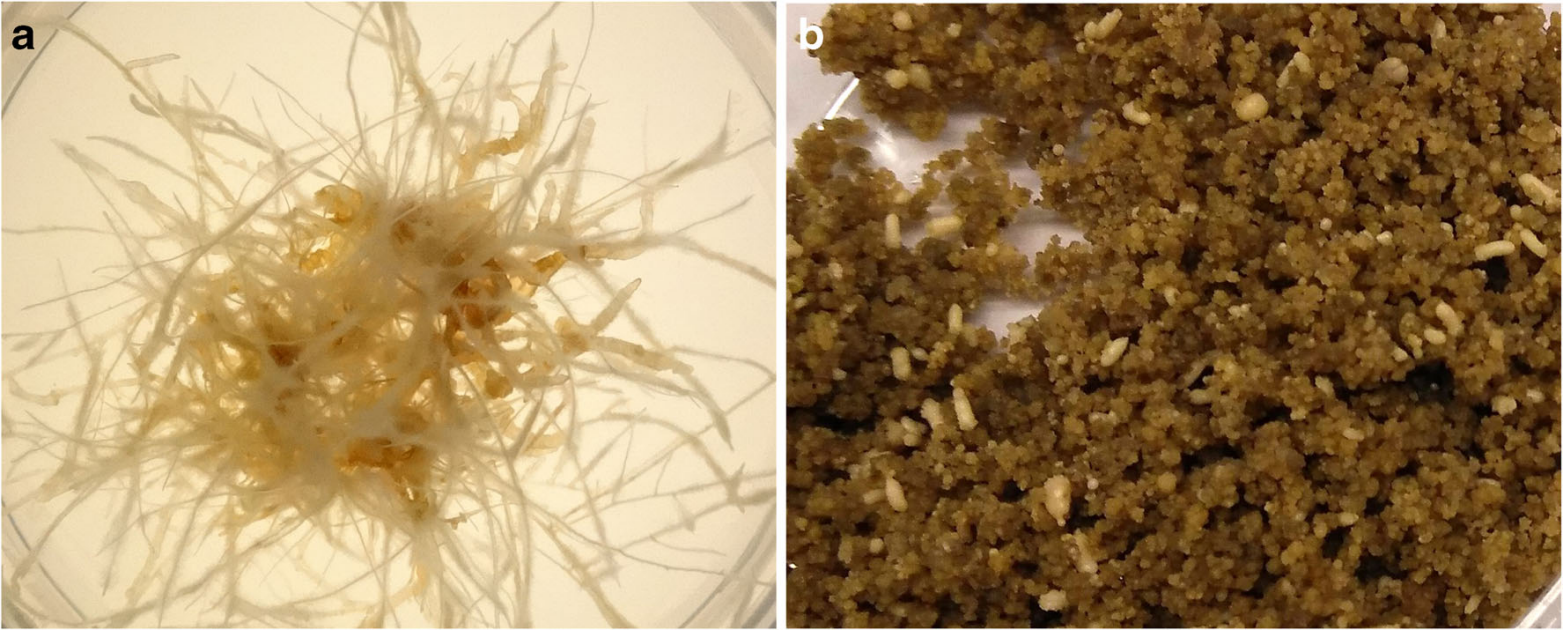
Examples of plant tissue cultures. **a** Hairy root culture of *Ocimum basilicum* (the culture was established at the Technical University Dresden, photo by Sibylle Kümmritz). **b** Somatic embryo culture of *Coffea canephora* (the culture was established at the Nestlé R&D Centre Tours as presented at the DECHEMA Himmelfahrtstagung 2018 in Magdeburg, Germany, poster contribution)

For cosmetics and food products, plant tissue cultures play a minor role in contrast to the previously described plant suspension cultures. Thus, we give this topic only marginal consideration. There are just a few product examples that are based on hairy roots (e.g., Mibelle Biochemistry’s RootBioTec HO from *Ocimum basilicum*) and somatic embryo cultures (e.g., Vitalab’s Vita Nova from *Lotus japonica*). Hairy roots (Fig. 2a) are generated following infection with the soil bacterium *Rhizobium rhizogenes* (formerly *Agrobacterium rhizogenes*), which shifts the transfer-DNA (T-DNA) originating from the root inducing plasmid (Ri plasmid) into the plant genome. The successful transformation process results in the formation of proliferating roots, so-called hairy roots, on the explant infection side. Hairy root cultures are characterized by lateral branching, similar growth to plant suspension cultures, a hormone-free propagation procedure, a lack of geotropism, and genetic stability. But hairy roots can only be applied to produce bioactive compounds synthesized within the roots of the parent plant. Detailed information about hairy root culture establishment, maintenance, and cultivation is provided by Georgiev et al. (2007), Eibl et al. (2009a), Pistelli et al. (2011), Sharma et al. (2013), and Sena (2015).

Somatic embryos delivering bioactive compounds are morphologically and physiologically identical to zygotic embryos present in the seeds of the parent plant. They are induced by either differentiated or undifferentiated somatic cells through a series of morphological and biochemical changes. The developmental processes of the somatic embryos are regulated by multiple factors, to which also phytohormones belong. For more detailed information, the interested reader is referred to Hess (1992), Quiroz-Figueroa et al. (2006), Tito et al. (2015), and Jang et al. (2016). Figure 2b shows a somatic embryo culture at the end of the somatic embryogenesis, known as torpedo-stage embryos.

## Plant cell culture propagation and extract manufacture

### Selection of the optimum cultivation parameters and the most suitable bioreactor type

The plant cell culture type, in particular, its morphology, growth, and production behavior, influences the selection of the bioreactor type and the definition of its optimum cultivation parameters. By screening a highly productive production cell line, and optimizing the culture medium and environment, up to 30-fold increases in product titers have been reported (Ochoa-Villarreal et al. 2015).

A further stimulation of the secondary metabolism is achievable by illumination with light (Curtin et al. 2003; Cuperus et al. 2007; Tassoni et al. 2012) or/and elicitation (Naik and Al-Khayri 2016; Sevón 1997; Lijavetzky et al. 2008; Goel et al. 2011; Zhou et al. 2015). The agents used for elicitation, the so-called elicitors, bind to specific receptors on the outside of the cytomembrane of the plant cell and trigger signaling cascades, which activate transcription of genes for synthesis of phytoalexins, reactive oxygen compounds, and defense enzymes. According to their origin, we distinguish between biotic elicitors (e.g., cell wall and membrane compounds, glycoproteins, modified nucleic acids) and abiotic elicitors (e.g., ultraviolet radiation, heavy metals, heat, cold). But establishing an effective elicitation process requires determination of the optimal elicitor type, dosage, and exposure time and, thus, is very laborious. Nevertheless, elicitation has been widely applied to increase the production of plant cell culture-based secondary metabolites (Singh and Dwivedi 2018) and is regarded as most effective approach. As shown by Jeandet et al., grape suspension cell culture-based production of resveratrol is feasible in bioreactors with titers up to 7 g L^−1^ when the production process is induced by the combination of methyl jasmonate and a cyclodextrin (Jeandet et al. 2016). This is the highest product titer reported in a plant cell culture-based secondary metabolite production process so far. Above all, elicitation may even cause the secretion of secondary metabolites, which typically are intracellular products.

Nowadays, the user can choose from numerous different bioreactor types that have been used in cultivations with plant cell cultures over decades. The selection of the bioreactor type most suitable for a particular bioprocess is a very complex task, as shown by Werner et al. for plant cell cultures. The optimum bioreactor type should be well-instrumented as well as scalable and should support the growth of the production cell line and the formation of the desired bioactive compound(s) while keeping the bioreactor footprint low. This means that mixing gas supply and dispersion of the plant cell culture have to be sufficient, while mass transfer limitations and accumulations of harmful by-products should be avoided (Werner et al. 2017). Excessively high shear forces in the bioreactor, which result from high specific power input by mixing and/or aeration, need to be excluded. Homogeneous and sufficient illumination and dissipation of the heat for photoauto- and photomixotroph plant cell cultures in the bioreactor are also required. However, demands in terms of the tolerable oxygen transfer rate as well as power input and the intensity (80.7–1345 μmol m^−2^ s^−1^) and duration (0, 8, 16, 24 h) of illumination may differ for cell growth and product formation (Chattopadhyay et al. 2002; Eibl et al. 2009a; Hasan et al. 2017). Werner et al. (2017) propose selecting the bioreactor type depending on the most suitable volumetric oxygen transfer coefficient (k_L_a) to specific power input (P/V) ratio in a first step. Subsequently, the design of the bioreactor type (e.g., impeller type and number, sparger type) can be modified if required and possible. Usage of computational fluid dynamics (CFD) has been shown to be beneficial for the optimization of bioreactor design and the definition of the main process parameters (e.g., impeller speed, rocking rate, rocking angel, aeration rate) to be realized. In principle, it is possible to more rapidly develop and manufacture bioreactor prototypes and to reduce the number of experiments by applying CFD (Werner et al. 2014b).

Plant cell suspension lines, in particular those growing slowly and behaving as Newtonian fluids, are very easy to propagate in bioreactors. Attention must be paid to enabling sufficient power input and oxygen when plant cell cultures with non-Newtonian fluid flow behavior (usually fast growing plant cell suspension cultures) are to be proliferated without damage by hydromechanical stress (Eibl et al. 2009b; Werner et al. 2014a). As a general rule, plant cell suspension cells with slow or moderate growth can be propagated in the same bioreactor types as mammalian suspension cells. The oxygen demand is comparable, maximum oxygen uptake rates of between 2 and 10 mmol L^−1^ h^−1^ having been determined for plant suspension cells (Curtis et al. 2006). But the majority of the plant suspension cells tolerate higher hydromechanical stress than mammalian suspension cells (Eibl et al. 2009a). An even greater challenge than the cultivation of plant suspension cells, characterized by a slow to moderate growth in bioreactors, is that of fast growing plant suspension cells, hairy root and somatic embryo cultures. It is a matter of fact that the cultivation of fast growing suspension cell lines is often accompanied by strong foam formation and flotation. This does not apply to hairy root and somatic embryo cultures, where bioreactor types guaranteeing homogeneous power input, oxygen, and light supply as well as avoiding high shear stress peaks are preferred.

### Most frequently used bioreactor types

**Fig. 3:**
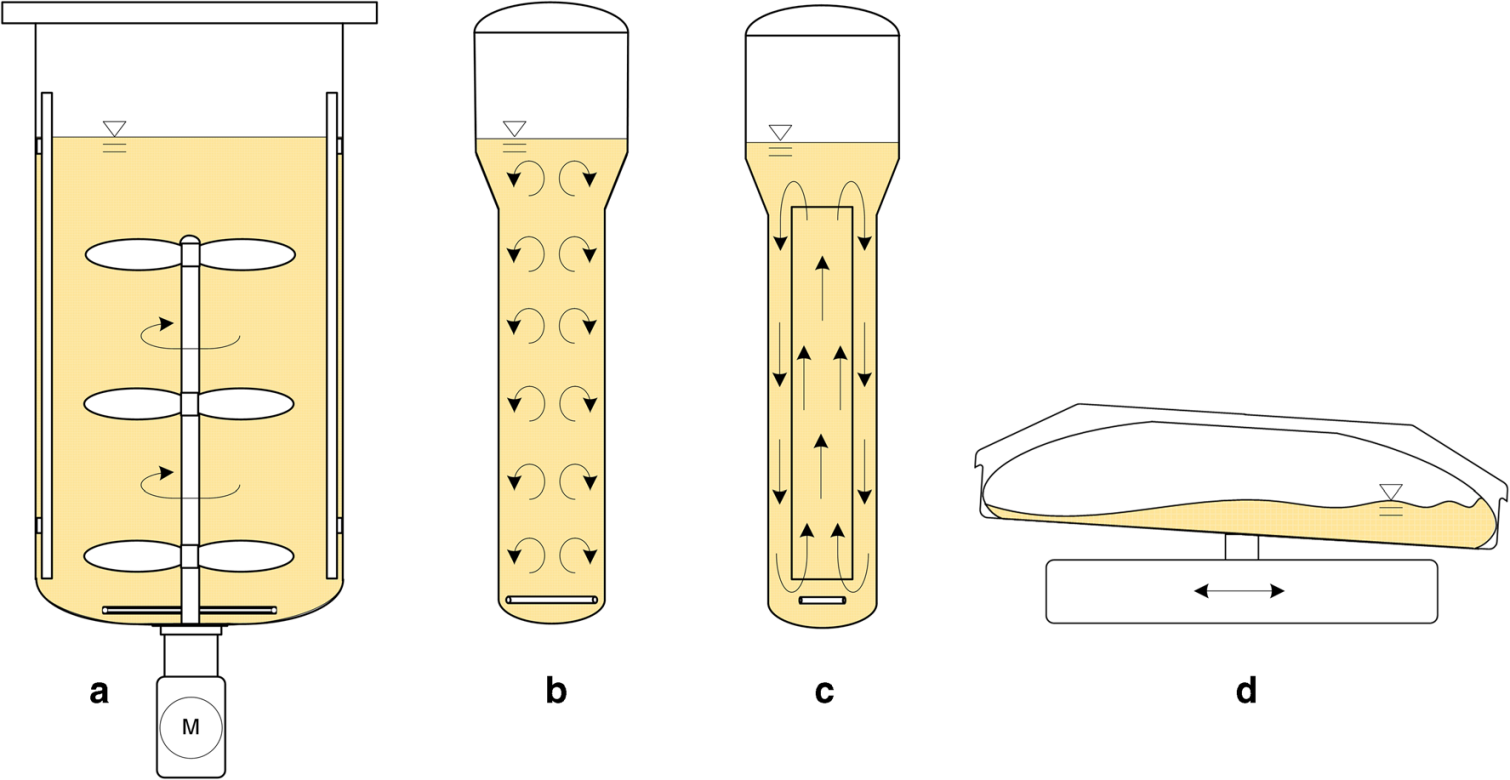
Schematic diagrams of instrumented bioreactors preferred in commercial production processes with plant cell cultures which generate products for the cosmetics and food industries. **a** Stirred bioreactor. **b** Bubble column bioreactor. **c** Airlift bioreactor. **d** Wave-mixed bioreactor with 1-D motion

Nowadays, stirred bioreactors, bubble column bioreactors, airlift bioreactors, and wave-mixed bioreactors with one-dimensional (1-D) motion are most often used for commercial productions with plant cell cultures (Ruffoni et al. 2010; Georgiev et al. 2013; Steingroewer et al. 2013; Stiles and Liu 2013; Imseng et al. 2014; Lehmann et al. 2014; Mamun et al. 2015). The working principles of these four bioreactor types are depicted in Fig. 3. When focusing on the cubic meter scale, stainless steel stirred bioreactors (Fig. 3a) prevail. They belong to mechanically driven bioreactors and are commonly regarded as the system of choice for plant cell suspension cultures. In contrast to their stirred counterparts, reusable bubble column (Fig. 3b) and airlift bioreactors (Fig. 3c) have no moving parts. They are pneumatically driven and are used to mass propagate the more shear sensitive plant tissue cultures (hairy root and somatic embryo cultures) up to cubic meter scale. Bioreactor modifications, such as an increase in diameter of the bioreactor head section to contribute to foam reduction, have been made (Paek et al. 2005; Jiang et al. 2015). Such bubble column and airlift bioreactors with a balloon-like head section are often referred to as “balloon-type systems.”

Where working volumes up to 300 L are sufficient, wave-mixed bioreactors with 1-D motion (Fig. 3d) are frequently operated with plant cell cultures. This bioreactor type belongs to the group of so-called single-use bioreactors. The wave-mixed bioreactor with 1-D motion has a multilayer plastic bag as a cultivation container. The bag is provided ready-to-use by the bioreactor supplier and is discarded after one single usage. Lehmann et al. (2014) present single-use bioreactors suitable for plant cell cultures in their review. Furthermore, they have compiled bioengineering data for wave-mixed bioreactors with 1-D motion. These bioreactors are obtainable from different suppliers at up to 600 L total volume and may even be equipped with light-emitting diodes. By moving the rocker unit, a wave is induced in the bag containing the culture medium and cells. In this way, mixing occurs and surface aeration takes place while the medium surface is permanently renewed. The power input is controllable by the rocking angle, rocking rate, and filling level of the bag. Wave-mixed bioreactors with 1-D motion are suitable for both plant cell suspension cells and tissue cultures. Normally, an antifoam agent is not required, because the foam is constantly incorporated into the culture broth. At high cell densities and culture broth viscosities, mass transfer can be limited in wave-mixed bioreactors with 1-D motion. A solution might be the application of a wave-mixed bioreactor with multi-dimensional motion such as the Cell tainer (Oosterhuis and Junne 2016). However, no reports about its usage for commercial cultivations of plant cell cultures have appeared in the literature to date.

In addition, there are a high number of further suitable bioreactor types for plant cell cultures. However, they are partially non-instrumented and have been used only for research purposes or displayed in-house developments of research groups and companies. Examples are temporary immersion systems (Ducos et al. 2007; Ducos et al. 2010; Georgiev et al. 2014), rotating drum bioreactors (Tanaka et al. 1983; Georgiev et al. 2013), mist bioreactors (Weathers et al. 2008; Weathers et al. 2010; Fei and Weathers 2014), and bioreactor systems with orbitally shaken bags (Werner et al. 2013; Lehmann et al. 2014).

### Plant cell culture extract manufacture

The process of extract manufacture following cultivation in a bioreactor is highly dependent on the chemical nature(s) of the bioactive substance(s) to be contained in the plant cell culture extract. Moreover, whether the extract is a liquid or a powder needs to be taken into account. A distinction is made between hydrosoluble (e.g., amino acids, glucides, flavonoids, anthocyanins, phenolic acids) and liposoluble (e.g., vitamins, tocopherols, fatty acids) compounds and those derived from plant cell walls (mixtures of peptides and sugars). Independent of the extract type, the first step is always to harvest the culture broth containing the intracellular target compound(s). The subsequent operations are manufacturer specific and generally include harvest, homogenization and disruption of the cell mass, extraction with solvents or proteolytic enzymes and/or chromatographic methods, and washing steps (Venkatramesh et al. 2010; Barbulova et al. 2014; Morus et al. 2014). Furthermore, if the extract is a powder, a drying process with freeze dryers, spray dryers, or vacuum dryers is required. There are also manufacturing processes for plant cell culture extracts that differ from the above, the details of which can be found in the manufacturers’ patent documents. One example is Mibelle Biochemistry’s process for extract manufacture of the PhytoCELLTECH actives (Table 1). After a mixing process with liposomes, phenoxyethanol, and antioxidants (ascorbic acid or tocopherol), a liquid extract is produced from all the compounds, including plant cells that have been disrupted by high-pressure homogenization at 1500 bar (Blum et al. 2013).

It is important that plant cell culture extracts for the cosmetics and food industries are not of toxicological concern when it comes to their final use. In the case of supplement ingredients contained in very low concentrations in the final product, a risk scenario for the consumer is rather unlikely. In cosmetics products, for example, the levels are often below 1%. The replacement of traditional, synthetic phytohormones in the culture medium (2.4-dichlorophenoxyacetic acid, 6-bezylaminopurine, N6-furfuryladenine) with natural phytohormones (indole-3-acetic acid, zeatin) and the application of phytohormone elicitors (jasmonic acid, methyl jasmonate, salicylic acid) and/or light further increase the safety of plant cell culture-based products for the cosmetics and food industries (Murthy et al. 2015). However, more attention needs to be paid to plant cell culture-based food supplements and food. When the plant cell culture is the foodstuff itself, not only does a risk analysis have to be carried out by a toxicologist but a food-conform culture medium is also necessary.

## Latest developments in plant cell culture-based food production

### Example 1: cell culture chocolate

The megatrends of society, health, individualization, and mobility have effects on consumer behavior in terms of food culture and resulting developments in the food industry (Berghofer et al. 2015; Reynolds 2016). According to the recent European Food Trends Report of the Gottlieb Duttweiler Institute, there are two main trends, a healthy way of eating and high tech food (Schäfer et al. 2017). Customers want to be actively involved in the production, distribution and consumption of their food. Plant cell cultures provide innovative solutions in this context. As mentioned above, their establishment is independent of location, and their metabolism can effectively be influenced (e.g., by medium composition and/or cultivation parameters) during the mass propagation procedure. On the one hand, it is possible to produce bioactive compounds which are responsible for food aroma and taste and/or have health benefits. On the other hand, the formation of harmful compounds can be reduced or completely suppressed by working with plant cell cultures.

**Fig. 4:**
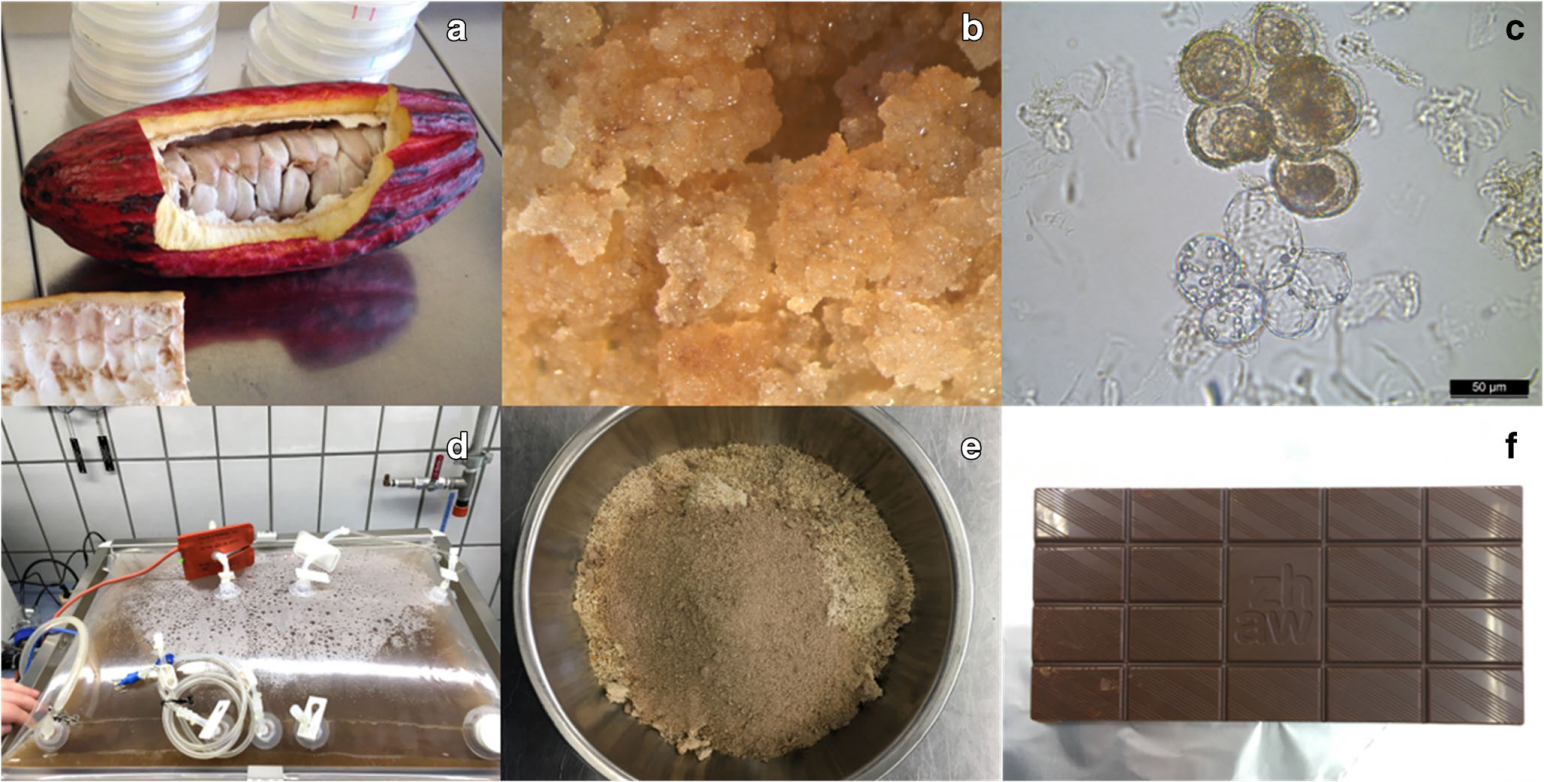
Main steps of the production of chocolate based on *Theobroma cacao* suspension cells. **a** One of the cacao fruits used to induce seven callus culture cell lines from beans. **b** Established callus culture of the clone ICS-45. **c** Microscopic picture of *T. cacao* suspension cells growing in shake flasks (clone ICS-45). **d** Twenty-L Flexsafe bag with mass propagated *T. cacao* suspension cells. **e** *T. cacao* suspension cells after freeze drying. **f** Produced cell culture chocolate

Such an approach has been implemented in a Zurich University of Applied Sciences (ZHAW) study which investigated the potential of callus and suspension cell lines of *Theobroma cacao* for the cocoa ingredient in chocolate production. In contrast to the developments published by Diana Plant Sciences (Venkatramesh et al. 2010; Barney 2013), where the cell cultures originated from immature *T. cacao* floral explants, cocoa beans (seeds; Fig. 4a) of cocoa fruits (origin: USDA-ARS Tropical Agriculture Research Station in Puerto Rico, 2200 P.A. Campos Ave., Suite 201, Mayaguez, Puerto Rico) characterized by different stages of maturity were used in our lab (Stutz 2018). Four callus cell lines grown at 29 °C on a modified Murashige and Skoog medium in the dark reached doubling times of 7 days. Whereas these callus cultures had comparable or even up to 40% higher polyphenol content (epicatechin, procyanidins B1, B2, C1, and cinnamtannin A2) than the source material (*T. cacao* beans), the alkaloids caffeine and theobromine were reduced by up to 100%. Furthermore, an overexpression of the amino acids valine, cysteine, and phenylalanine, which are known to boost the immune system, was detected. Because the callus clone ICS-45 (Fig. 4b) was also very friable, it was decided to generate the production suspension cell line from it (Fig. 4c). The *T. cacao* suspension cells reached doubling times of 4 days when propagated in 1-L shake flasks over 9 days in batch mode (300 mL working volume, 29 °C, 120 rpm, 25 mm shaker amplitude). The single cells of *T. cacao* had a spherical shape and a diameter between 25 and 50 μm, which is about 50% smaller than usual. With increasing cultivation time, the *T. cacao* suspension cells propagated in a modified Murashige and Skoog medium tended to grow in large clusters, the vast majority of which appeared slightly brownish under the microscope.

Based on the results of the shake flask runs, the process was transferred to a wave-mixed single use bioreactor in order to generate sufficient *T. cacao* cell mass for the production of cell culture chocolate. We worked with Sartorius Stedim’s BIOSTAT RM 20/50 equipped with a 20-L bag with screw cap (Fig. 4d). A feed with 5-L medium was realized on day 7 (initial working volume 6 L). About 300 g biomass (fresh weight) was harvested on day 16, separated from the culture broth, rinsed, and freeze-dried (Fig. 4e). The in vitro cocoa powder was used to produce three bars of 70% dark chocolate (Fig. 4f). While the biomass was not pre-treated for the first bar, the biomass of the second bar was completely aerobically incubated (46 h) and that for the third bar both anaerobically (30 h) and aerobically (16 h) incubated. The purpose of this procedure was to simulate the fermentation of the cocoa beans, which is crucial for the development of aroma in the traditional production of chocolate. The cocoa powder was roasted, sugar and cocoa butter were added, and the mixture was rolled. Before lecithin was added, the chocolate mass was heated up. Finally, the chocolate mass was casted into forms. Professional chocolate tasting was carried out by a ZHAW expert panel, who confirmed that the cell culture chocolate provided a unique taste experience. Interestingly, the untreated bar of chocolate performed best. An intense and complex aroma was described, whereby citrus and berry aromas were predominant. Beyond that, lactic, malty and green tones, and a mildly acidic component were detected in the profile. The different expressions were also confirmed by a first aroma analysis (volatile aroma compounds). However, our investigations are still ongoing, and future studies will include, for example, the increase in process efficiency.

### Example 2: plant cell culture-based foodstuff produced in a home bioreactor

**Fig. 5:**
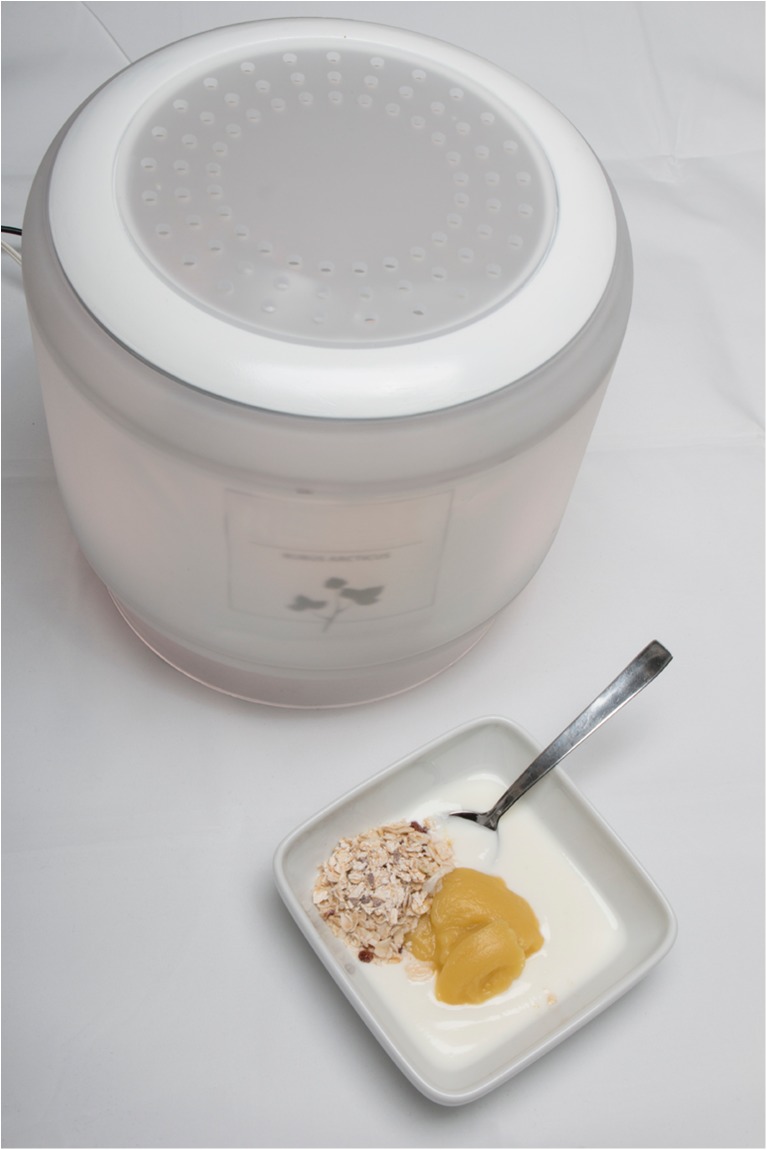
VTT’s home bioreactor, which was developed in co-operation with designers at the Aaolto University School of Arts, Design and Architecture, and which may be applied to produce berry cell culture biomass for the morning-cereal or smoothie, or which can be eaten as supplement in the future (with kind permission of Dr. Heiko Rischer, VTT Technical Research Centre of Finland Ltd.)

Another interesting approach for modern plant cell culture-based food production is used by specialists from the Technical Research Center of Finland Ltd. (VTT), who developed a bioreactor to produce about 500 g (fresh weight) of edible plant cell culture biomass within 1 week at home. The bioreactor, referred to as “Home bioreactor” and working in a similar way to a coffee machine, is shown in Fig. 5. Its current design is about the size of a table lamp and consists of a container with a lid, which has been manufactured with a three-dimensional printer. The bioreactor container has two openings: one for the insertion of a single-use bag or capsule with the cell starter (plant cell culture with medium) and one for adding water. By turning on the bioreactor, whose temperature is controlled, and which can be illuminated and aerated, the cell culture is kept at optimal growth conditions (Räty 2017). Although this bioreactor, which was listed among the ten Forbes’s food trends of 2017 (Lempert 2016), has already been successfully applied to mass propagate suspension cultures of blackberries and cloudberries in first tests, it is not yet ready for the market. Moreover, additional questions concerning sterility, cell culture inoculum (stable, easy to handle, full-bodied taste), and culture medium (food-conform, cheap) have to be addressed.

All in all, it is expected that this bioreactor, including cell cultures and appropriate culture media, will be available on the market within the next 9 years. The idea of cooking with plant cell cultures that have been mass propagated in consumers’ own kitchens should then become a reality.

## Conclusions

The use of plant cell cultures instead of whole plants allows products for the cosmetics and food industries to be manufactured with less energy, lower possible impacts on the environment, and independent of location and season. The currently available products are supplement ingredients to reduce hair loss and aging of skin, which improve skin quality and strengthen the body’s immune defense system. In today’s commercial manufacture, plant cell suspension cultures are grown in reusable stainless steel stirred bioreactors or single-use wave-mixed bioreactors with 1D-motion for the most part. Due to small working volumes, higher safety, more rapid and simple putting into operation, and shorter development times, single-use wave-mixed bioreactors are ideal for research and development and for personalized products. However, with very few exceptions, these bioreactors were originally designed for pharmaceutical high-value products based on mammalian cell cultures. The demand for bioreactors providing bioactive substances for the pharmaceutical industry is higher than in the cosmetics and food industries, where the lower demand for plant cell culture-based productions of cosmetics and food supplement ingredients means that they are often too expensive.

In line with the current trend for home-made food, there is a need for low-cost bioreactors which are easy to handle and provide cell mass in the three-digit and four-digit g-range. The Finnish “Home bioreactor” is a first approach but has not yet been commercialized and made suitable for everyday use. In addition to this novel bioreactor, it should be ensured that the user has access to inoculum cultures and suitable food-compatible culture media in the future. Indeed, it seems that the way to a food revolution has been paved and plant cell culture-based superfood independent of location will soon be obtainable.
